# Water-accelerated π-Stacking Reaction in Benzene Cluster Cation

**DOI:** 10.1038/s41598-019-39319-7

**Published:** 2019-02-20

**Authors:** Hiroto Tachikawa, Ryoshu Iura, Hiroshi Kawabata

**Affiliations:** Division of Applied Chemistry, Graduate School of Engineering, Hokkaido University, Sapporo, 060-8628 Japan

## Abstract

Single molecule electron devices (SMEDs) have been widely studied through both experiments and theoretical calculations because they exhibit certain specific properties that general macromolecules do not possess. In actual SMED systems, a residual water molecule strongly affects the electronic properties of the SMED, even if only one water molecule is present. However, information about the effect of H_2_O molecules on the electronic properties of SMEDs is quite limited. In the present study, the effect of H_2_O on the ON-OFF switching property of benzene-based molecular devices was investigated by means of a direct ab initio molecular dynamics (AIMD) method. T- and H-shaped benzene dimers and trimers were examined as molecular devices. The present calculations showed that a H_2_O molecule accelerates the π-stacking formation in benzene molecular electronic systems. The times of stacking formation in a benzene dimer cation (*n* = 2) were calculated to be 460 fs (*H*_2_*O*) and 947 fs (*no-H*_2_*O*), while those in a trimer cation (*n* = 3) were 551 fs (*H*_2_*O*) and 1019 fs (*no-H*_2_*O*) as an average of the reaction time. This tendency was not dependent on the levels of theory used. Thus, H_2_O produced positive effects in benzene-based molecular electronics. The mechanism of π-stacking was discussed based on the theoretical results.

## Introduction

Molecular electronics is a new field of technology that is based on the applications of electronic devices composed of organic molecules^1–7^. Although a variety of functional molecular devices such as molecular switches^8–10^ and molecular rectifiers^11,12^ have been prepared successfully, the field of molecular electronics is still very fertile from a fundamental research perspective, allowing rapid progress to be made in device performance and reliability.

Among molecular electronic devices, single molecule (including small cluster)-electron devices (SMEDs) have been widely investigated, by experiments and theoretical calculations, in view of certain specific properties that general macromolecules do not possess. For instance, slight changes in the molecular structure and constituent atoms of SMEDs can lead to large differences in electron conductivity. Using density functional theory (DFT) calculations, Yang *et al*. investigated the effect of atom substitution on the electron transport properties in dehydroazulene^13^. They suggested that different substitution positions of fluorine atoms in the molecule had a significant influence on the switching property.

In actual SMED systems, a residual water molecule may strongly affect the electronic properties, even if only one water molecule is present. However, information about the effect of H_2_O molecules on the electronic properties of SMEDs is quite limited. Recently, Li *et al*. investigated the effect of H_2_O on the conductance of a single molecular junction composed of thiolated arylethynylene with a 9,10-dihydroanthracene core (denoted as TADHA)^14^. These authors showed that H_2_O suppressed the conductance of the molecular junction if they adsorbed on the terminal sulfur atoms. The circuit of electron transport in Na-NTCDA (1,4,5,8-naphthalene-tetracarboxylic-dianhydride) was easily destroyed by a single water molecule^15^. In molecular devices comprising biphenyl molecules^16^, H_2_O strongly affected the hole transport properties. Thus, previous reports have shown that the H_2_O molecule generally produces negative effects on molecular electronics^17–20^.

In the present study, the effect of a single water molecule on the ON-OFF switching property of benzene-based molecular devices was investigated using the direct ab initio molecular dynamics (AIMD) method^21–23^. The small-sized benzene clusters have the ability to act as an ON-OFF switching element^24–26^. It is known that a T-shaped benzene dimer is drastically changed to the π-stacking form after hole capture, while an H-shaped benzene trimer is changed to the double π-stacking form. In this work, we mainly focused on the effect of H_2_O on the time-scale of π-stacking formation in benzene clusters.

## Computational Details

### Static density functional theory (DFT) calculations

The geometries of a T-shaped benzene dimer, H-shaped benzene trimer, hydrated benzene dimer, and hydrated benzene trimer were fully optimized using the CAM-B3LYP/6-311++G(d,p) method. The atomic and molecular charges were calculated using natural population analysis (NPA). The standard Gaussian 09 program package was used for all static ab initio calculations^27^.

### Direct AIMD calculations

The trajectory of (Bz)_n_^+^ following the ionization of (Bz)_n_ (*n* = 2) was calculated using direct AIMD^21–23^ at the CAM-B3LYP/6-31 G(d) level under the assumption of vertical ionization in the neutral state. The optimized structure obtained at the CAM-B3LYP/6-311++G(d,p) level was chosen as the initial geometry of (Bz)_n_^+^ at time zero. The trajectory calculation of (Bz)_n_^+^ was performed using the condition of constant total energy. The velocity Verlet algorithm was used with a time step of 0.5 fs to solve the equation of motion for the system. The drifts in the total energies in all trajectory calculations were less than 0.01 kcal/mol. Similar calculations were carried out for hydrated benzene dimer and trimer systems.

To investigate the effect of the initial structures on the reaction mechanism and time-scale of stacking formation in (Bz)_2_^+^ and (Bz)_2_^+^-H_2_O, the initial geometries were generated by direct AIMD calculations under a constant temperature condition^28,29^. The temperature was chosen as 10 K. First, direct AIMD calculations of the neutral systems, (Bz)_2_ and (Bz)_2_-H_2_O, were carried out at the CAM-B3LYP/6-311++G(d,p) level at 10 K. Second, the geometries and velocities of the atoms were selected from the simulations. Next, direct AIMD calculations were carried out for cation systems at the CAM-B3LYP/6-31G(d) level. Twelve trajectories were run from the selected points.

In addition to the 10 K simulation, the optimized structures of the cations calculated by several levels of theory were examined as the initial structures in the direct AIMD calculations. The basis sets used were (A) 6-311++G(2d,p), (B) 6-311++G(2d,2p), (C) 6-311++G(2df,2p), (D) 6-311++G(2df,2pd), (E) 6-311++G(3df,2pd), and (F) 6-311++G(3df,3pd). The trajectories began from these optimized structures, and the times of π-stacking formation were calculated.

The dependence of the reaction time on the functional used for DFT calculations was assessed by using APFD, B3LYP, M052X, and M062X functionals. Note that the dependence on the type of functional was significantly small, as shown in later sections.

## Results

### Electronic states of (Bz)_n_ and (Bz)_n_-H_2_O (*n* = 2 and 3)

**Figure 1:**
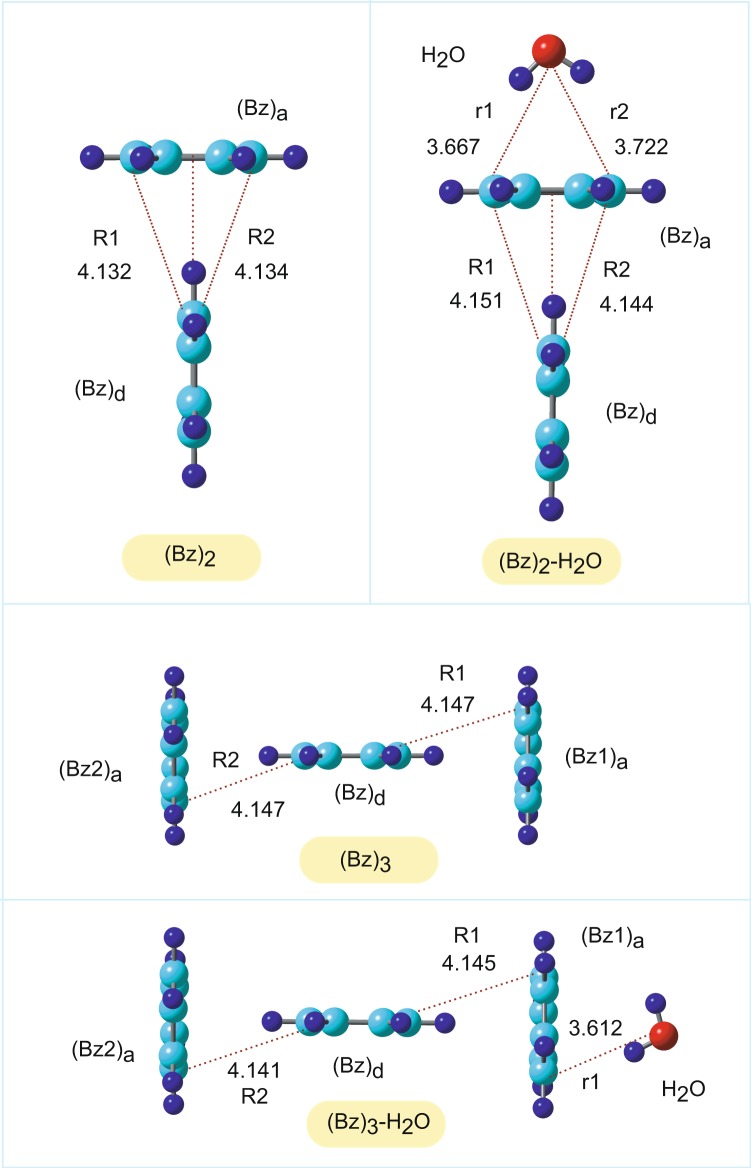
Structural and geometrical parameters of the benzene dimer (Bz)_2_ and trimer (Bz)_3_. (A) Optimized structures of (Bz)_2_, (Bz)_2_-H_2_O, (Bz)_3_, and (Bz)_3_-H_2_O calculated at the CAM-B3LYP/6-311++G(d,p) level. The values indicate the intermolecular distances (in Å).

The structures of the benzene (Bz)_n_ and hydrated benzene (Bz)_n_-H_2_O dimers and trimers (*n* = 2 and 3) were fully optimized at the CAM-B3LYP/6-311++G(d,p) level. The optimized structures are given in Fig. 1. The benzene dimer (*n* = 2) is composed of proton donor and acceptor benzene molecules, which are denoted as (Bz)_d_ and (Bz)_a_, respectively. The intermolecular carbon-carbon distances (Å) were (R1, R2) = (4.132, 4.134) in (Bz)_2_ and (4.151, 4.144) in (Bz)_2_-H_2_O, indicating that the effect of H_2_O on the structure was negligibly small, although the distance increased slightly as a result of the interaction with H_2_O. In (Bz)_2_-H_2_O, the dipole of H_2_O was oriented toward the center of mass in (Bz)_a_, while the distances of H_2_O from (Bz)_a_ (in Å) were (r_1_, r_2_) = (3.667, 3.722).

The NPA charges are given in Table 1. The NPA molecular charges on (Bz)_d_ and (Bz)_a_ in (Bz)_2_ were −0.002 and +0.002, respectively, indicating that the electron transfer from (Bz)_a_ to (Bz)_d_ was negligibly small. In the case of (Bz)_2_-H_2_O, the NPA molecular charges on (Bz)_d_, (Bz)_a_, and H_2_O were −0.002, 0.000, and +0.002, respectively. The charge distribution on the neutral benzene dimer was nearly unaffected by the interaction with H_2_O.

**Table 1:** NPA molecular charges and spin densities in (Bz)_2_, (Bz)_2_-H_2_O, [(Bz)_2_^+^]_ver_, and [(Bz)_2_^+^-H_2_O^+^]_ver_. The values were calculated at the CAM-B3LYP/6-311++G(d,p) level.

	System	(Bz)_d_	(Bz)_a_	H_2_O
NPA	(Bz)_2_	−0.002	+0.002	—
	(Bz)_2_-H_2_O	−0.002	0.000	+0.002
	[(Bz)_2_^+^]_ver_	+0.659	+0.341	—
	[(Bz)_2_^+^-H_2_O]_ver_	+0.818	+0.177	+0.005
spin dens.	[(Bz)_2_^+^]_ver_	0.661	0.339	—
	[(Bz)_2_^+^-H_2_O^+^]_ver_	0.821	0.181	−0.001

The benzene trimer (*n* = 3) is composed of one proton donor and two acceptor benzene molecules, which are denoted as (Bz)_d_, (Bz1)_a_, and (Bz2)_a_, respectively. The intermolecular distances (R1, R2) were (4.147, 4.147) in (Bz)_3_ and (4.145, 4.141) in (Bz)_3_-H_2_O. The dipole of H_2_O orients benzene (Bz1)_a_. The NPA charges of (Bz)_3_ and (Bz)_3_-H_2_O are given in Table 2. The effects of H_2_O on the electronic states were negligibly small in the neutral state for *n* = 2 and 3.

**Table 2:** NPA molecular charges and spin densities in (Bz)_3_, (Bz)_3_-H_2_O, [(Bz)_3_^+^]_ver_, and [(Bz)_3_^+^-H_2_O]_ver_. The values were calculated at the CAM-B3LYP/6-311++G(d,p) level.

	System	(Bz)_d_	(Bz1)_a_	(Bz2)_a_	H_2_O
NPA	(Bz)_3_	−0.004	+0.002	+0.002	—
	(Bz)_3_-H_2_O	−0.004	0.000	+0.002	+0.002
	[(Bz)_3_^+^]_ver_	+0.679	+0.161	+0.161	—
	[(Bz)_3_^+^-H_2_O]_ver_	+0.717	+0.043	+0.237	+0.003
spin dens.	[(Bz)_3_^+^]_ver_	0.684	0.158	0.158	—
	[(Bz)_3_^+^-H_2_O^+^]_ver_	0.721	0.044	0.235	0.00

### Electronic states at the vertical ionization points

Following the ionization, the reaction point was vertically shifted from the ground to ionization state. Hereafter, the vertical ionized states of (Bz)_n_ and (Bz)_n_-H_2_O are expressed as [(Bz)_n_^+^]_ver_ and [(Bz)_n_^+^-H_2_O]_ver_, (*n* = 2 and 3), respectively, where [X^+^]_ver_ indicates a radical cation of X at the vertical ionization point from its parent neutral species X.

In [(Bz)_2_^+^]_ver_, the NPA molecular charges on (Bz)_d_ and (Bz)_a_ were calculated to be +0.659 and +0.341, respectively, indicating that a positive charge was asymmetrically distributed on the benzene dimer cation (Table 1): the value of the charge on (Bz)_d_ was about two times larger than that of (Bz)_a_. In [(Bz)_2_-H_2_O^+^]_ver_, the charges changed to +0.818 in (Bz)_d_, +0.177 in (Bz)_a_, and +0.005 in H_2_O. The positive charge on (Bz)_d_ increased because of its interaction with H_2_O, whereas the positive charge on (Bz)_a_ was decreased by H_2_O. The magnitude of the asymmetry in the charge distribution on (Bz)_2_^+^ was enhanced by H_2_O.

The spatial distributions of the spin density of [(Bz)_2_^+^]_ver_ and [(Bz)_2_-H_2_O^+^]_ver_ are illustrated in Fig. S1. In [(Bz)_2_^+^]_ver_, the calculated spin densities on (Bz)_d_ and (Bz)_a_ were 0.661 and 0.339, respectively, indicating that the distribution of the unpaired electron on (Bz)_d_ was about twice as large as that of (Bz)_a_ (Table 1). In [(Bz)_2_^+^-H_2_O]_ver_, the spin densities were 0.821 on (Bz)_d_, 0.180 on (Bz)_a_, and −0.001 on H_2_O, indicating that the positive charge and spin density were pushed out from (Bz)_d_ to (Bz)_a_ by H_2_O because of the positive charge of the proton of H_2_O in [(Bz)_2_-H_2_O^+^]_ver_. Thus, the distribution of electrons was strongly influenced by H_2_O in the cation system, and the asymmetry of the spin density distribution was enhanced by H_2_O.

The NPA charge and spin densities of the trimer system (*n* = 3) are given in Table 2. In the vertical ionized states of (Bz)_3_, [(Bz)_3_^+^]_ver_, the NPA molecular charges on (Bz)_d_, (Bz1)_a_, and (Bz2)_a_ were calculated to be +0.679, +0.161, and +0.161, respectively, indicating that a positive charge was symmetrically distributed on the benzene trimer cation; the value of the charge on (Bz)_d_ was about four times larger than that of (Bz)_a_.

In the vertical ionized states of (Bz)_3_-H_2_O, [(Bz)_2_-H_2_O^+^]_ver_, the charges were +0.717 in (Bz)_d_, +0.043 in (Bz1)_a_, +0.237 in (Bz2)_a_, and +0.003 in H_2_O. The magnitudes of the positive charges on (Bz)_d_ and (Bz2)_a_ were enhanced by interaction with H_2_O, whereas the positive charge on (Bz1)_a_ was decreased by H_2_O. In [(Bz)_3_^+^]_ver_, the spin densities on (Bz)_d_, (Bz1)_a_, and (Bz2)_a_ were 0.684, 0.158, and 0.158, respectively, indicating that the distribution of the unpaired electron on (Bz)_d_ was larger than those of (Bz1)_a_ and (Bz2)_a_. In [(Bz)_2_^+^-H_2_O]_ver_, the spin densities were 0.721 on (Bz)_d_, 0.044 on (Bz1)_a_, 0.235 on (Bz2)_a_, and −0.001 on H_2_O, indicating that the spin density was pushed out from (Bz)_d_ to (Bz)_a_ by H_2_O. This result suggested that H_2_O created asymmetry of the electronic structure in the radical cation state.

### π-Stacking formation in (Bz)_2_-H_2_O^+^

#### Potential energy

**Figure 2:**
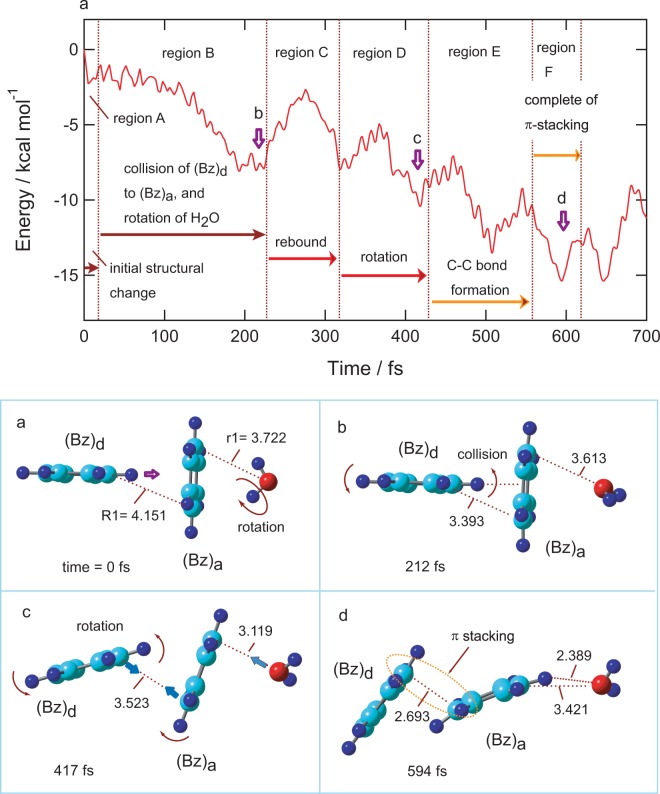
Results of direct AIMD calculation of (Bz)_2_^+^-H_2_O following the ionization of the parent neutral species. (Upper): Time evolution of the potential energy of (Bz)_2_^+^-H_2_O. (Lower): Snapshots of (Bz)_2_^+^-H_2_O after vertical ionization from the neutral state calculated as a function of time (intermolecular distances are in Å). The direct AIMD calculation was performed at the CAM-B3LYP/6-31G(d) level.

The time evolution of the potential energy of (Bz)_2_^+^-H_2_O, following the ionization of the parent neutral complex, is given in Fig. 2 (top). Also, the snapshots of (Bz)_2_^+^-H_2_O are shown in Fig. 2 (bottom). In this trajectory calculation, the optimized structure obtained at the CAM-B3LYP/6-311++G(d,p) level was used as the initial structure (time zero), and direct AIMD calculations were carried out at the CAM-B3LYP/6-31 G(d) level. The zero level on the vertical axis corresponds to the total energy of [(Bz)_2_^+^-H_2_O]_ver_ at time zero. The reaction dynamics of (Bz)_2_^+^-H_2_O could be classified by six regions as follows: region A: initial structural change, region B: collision of (Bz)_d_ to (Bz)_a_ and rotation of H_2_O, region C: rebound of (Bz)_d_ from (Bz)_a_, region D: rotation of (Bz)_d_ on (Bz)_a_, region E: C-C bond formation, and region F: complete of π-stacking.

After the ionization of (Bz)_2_-H_2_O (point a), the potential energy decreased suddenly to −2.0 kcal/mol within 5 fs. This energy decrease was caused by an internal structural deformation of (Bz)_2_^+^ after hole capture (region A). Then, the energy gradually decreased because of the changes in the structural conformation between (Bz)_d_ and (Bz)_a_ and the rotation of H2O on (Bz)2+. (Bz)_d_ gradually approached (Bz)_a_, and the collision of (Bz)_d_ to (Bz)_a_ occurred at 212 fs (point b in region B). In addition, the rotation of H_2_O occurred as a result of the repulsive interactions between the positive charges of the proton (H_2_O) and Bz^+^. The stabilization energy of the rotation of H_2_O was −4.0 kcal/mol, while the energy generated by collision of Bz^+^ with Bz was −2.0 kcal/mol. The internal structural deformation was −2.0 kcal/mol (0–5 fs). The total potential energy was −8.0 kcal/mol at point b. Moreover, the potential energy increased to −2.0 kcal/mol due to the rebound of (Bz)_d_ from (Bz)_a_ (region C). In conjunction with the rebound, the rotation of (Bz)_d_ on (Bz)_a_ occurred gradually, and the potential energy decreased again together with the vibration (region D). The snapshot at point **c** (417 fs) indicated that the structure of (Bz)_2_^+^ deformed gradually from a T-shape to the π-stacking form. In region E, the potential energy further decreased because a C-C bond was gradually formed between (Bz)_d_ and (Bz)_a_. At point d (594 fs in region F), the π-stacking formation was fully complete (final stage, region F). The potential energy reached a minimum point at 594 fs (point d). Thus, the time of π-stacking formation was calculated to be 594 fs in this trajectory.

#### Snapshots

Figure 2 (bottom) shows snapshots of (Bz)_2_^+^-H_2_O following the ionization of (Bz)_2_-H_2_O. The intermolecular distances between the benzene molecules were 4.151 Å for R1 and 3.722 Å for (Bz)_a_-H_2_O (r_1_). After ionization, the rotation of H_2_O on (Bz)_2_^+^ occurred, and (Bz)_d_ gradually approached (Bz)_a_ and collided at 212 fs (point b). After the collision, (Bz)_d_ rotated on (Bz)_a_. The arrows schematically indicate the direction of rotation. At 594 fs, the intermolecular distance was R1 = 2.693 Å and the π-stacking formation was complete.

It should be emphasized here that the H_2_O molecule showed a specific behavior after the ionization. The dipole of H_2_O oriented toward the center of mass of (Bz)_a_ at time zero (point a). After the ionization, H_2_O rotated rapidly on (Bz)_a_, and the oxygen atom of H_2_O interacted with (Bz)_a_ (regions A and B). After rotation, the oxygen atom of H_2_O oriented toward (Bz)_a_ (regions B-D). In region D, a CH-OH_2_ hydrogen bond was formed between (Bz)_a_ and H_2_O. In the final stage (point d in region F), the hydrogen bond and π-stacking formation were complete.

### π-Stacking formation in (Bz)_2_^+^ without H_2_O

**Figure 3:**
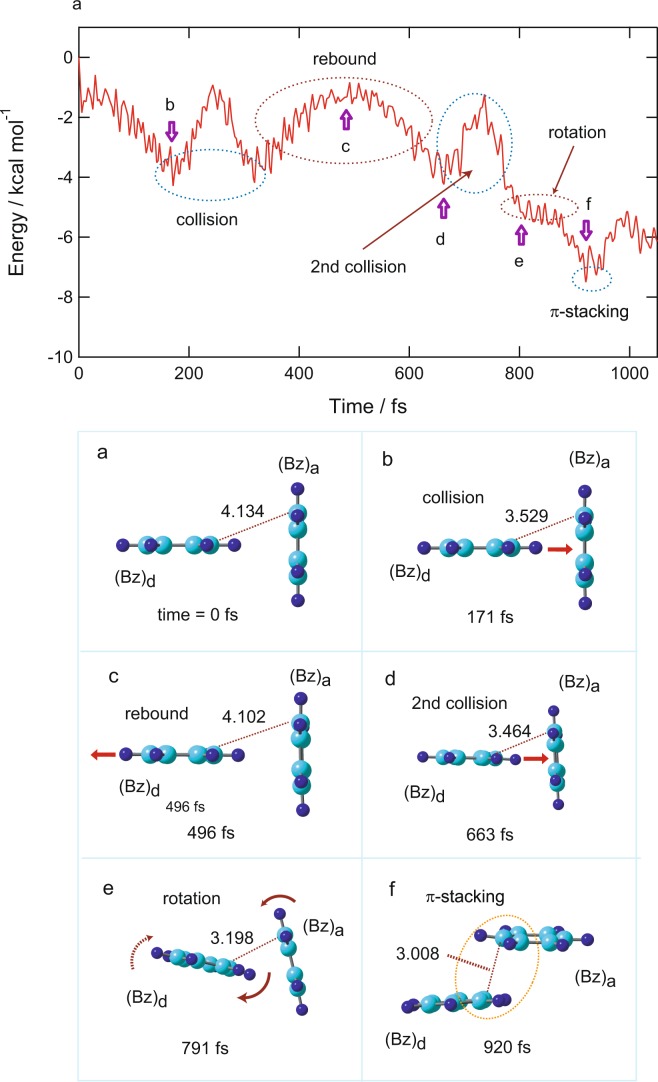
Results of direct AIMD calculation of (Bz)_2_^+^ following the ionization of the parent neutral species. (Upper): Time evolution of the potential energy of (Bz)_2_^+^. (Lower): Snapshots of (Bz)_2_^+^ after vertical ionization from the neutral state calculated as a function of time (intermolecular distances are in Å). The direct AIMD calculation was performed at the CAM-B3LYP/6-31G(d) level.

In the case of (Bz)_2_^+^-H_2_O, the π-stacking formation was complete at 594 fs. In this section, to elucidate the effects of H_2_O on the time of π-stacking formation, a similar direct AIMD calculation was carried out for the non-water system, (Bz)_2_. Figure 3 shows the potential energy and snapshots of (Bz)_2_^+^ following the ionization of (Bz)_2_. After the ionization, (Bz)_d_ gradually approached (Bz)_a_ and collided with (Bz)_a_ at 171 fs. The T-shaped structure was maintained at point c. (Bz)_d_ rebounded from (Bz)_a_ (496 fs, point c) and a second collision occurred at 663 fs (point d). After the second collision, both (Bz)_d_ and (Bz)_a_ rotated (791 fs, point e), and π-stacking was complete at 920 fs (point f). The time of π-stacking formation was 920 fs in this trajectory. The timescale for (Bz)_2_^+^ was about 300 fs longer than that of (Bz)_2_^+^-H_2_O, suggesting that one water molecule largely accelerated the π-stacking formation of (Bz)_2_.

### Effects of water molecule on time of π-stacking formation

**Figure 4:**
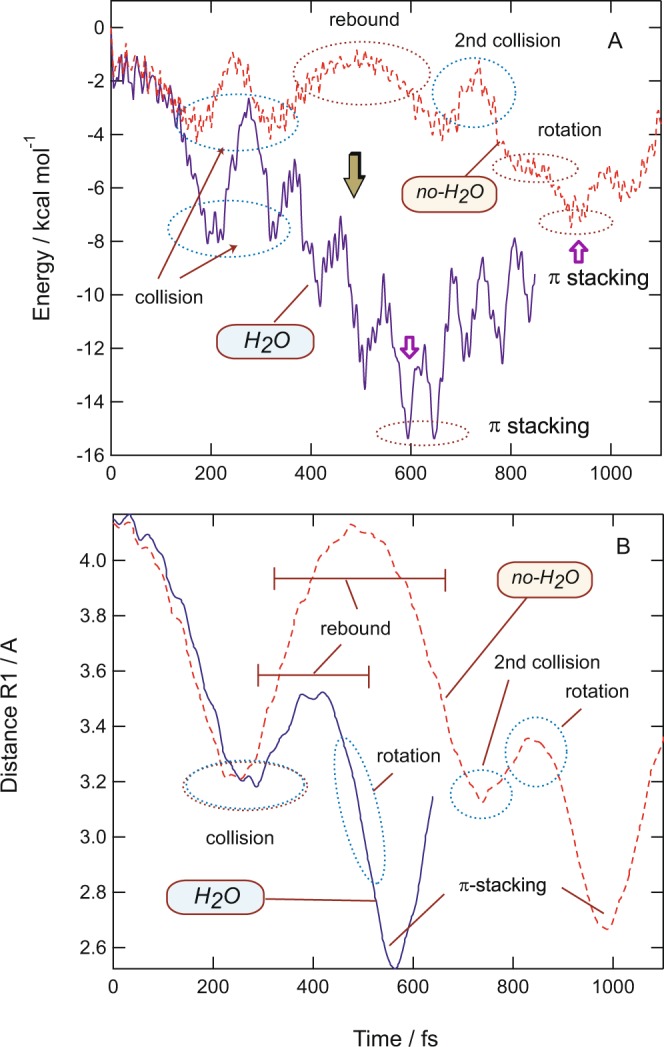
Comparison of reaction dynamics between (Bz)_2_^+^ (*no-H*_2_*O*) and (Bz)_2_^+^-H_2_O (*H*_2_*O*). (Upper): Time evolution of the potential energies of (Bz)_2_^+^ (*no-H*_2_*O*) and (Bz)_2_^+^-H_2_O (*H*_2_*O*). (Lower): Intermolecular distance calculated as a function of time (in Å). The direct AIMD calculations were performed at the CAM-B3LYP/6-31G(d) level.

In previous sections, it has been discussed that a water molecule accelerated the π-stacking formation in a benzene dimer cation. In this section, the reasons for the acceleration of the formation time by H_2_O are discussed based on theoretical results. Figure 4 shows the potential energies and intermolecular distances between the benzene rings plotted as a function of time for (Bz)_2_^+^ without H_2_O (denoted as *no-H*_2_*O*) and (Bz)_2_^+^-H_2_O (denoted as *H*_2_*O*). At 200 fs, the energy minima corresponding to the collision state were found in both systems. However, the values of energies were large different: −8.0 kcal/mol (*H*_2_*O*) and −4.0 kcal/mol (*no-H*_2_*O*). This difference was caused by the rotation of H_2_O on (Bz)_2_^+^. The rotation significantly stabilized the energy of the collision state of (Bz)_d_-(Bz)_a_^+^ because of the electrostatic effect.

After the collision, (Bz)_d_ rebounded from (Bz)_a_. In the case of *H*_2_*O*, the distance of (Bz)_d_ from (Bz)_a_ was calculated to be 3.5 Å at the turning point (400 fs), whereas the distance was 4.1 Å in *no-H*_2_*O* (480 fs). The distance at the turning point in *no-H*_2_*O* was 0.6 Å longer than that of *H*_2_*O*. In addition to the longer distance, the time in the bound state for *no-H*_2_*O* was significantly longer than that of *H*_2_*O* (200 vs. 350 fs). Also, the plateau of the potential energy caused by the rotation of (Bz)_d_ on (Bz)_a_ was found at 750–800 fs for *no-H*_2_*O*. In contrast, the rotation barrier disappeared in *H*_2_*O*. Thus, the effects of H_2_O on the reaction dynamics can be summarized as follows: (1) The excess energy caused by the rotation of H_2_O on (Bz)_2_^+^ contributed to acceleration of π-stacking formation, with the water molecule stabilizing the potential curve as a whole. (2) H_2_O could decrease the lifetimes of collision and rebound states as the oxygen atom of H_2_O attracts (Bz)_d_^+^ through electrostatic interactions. (3) The rotation barrier between Bz-Bz was also diminished by H_2_O.

### Effect of initial structure on time of π-stacking formation

**Figure 5:**
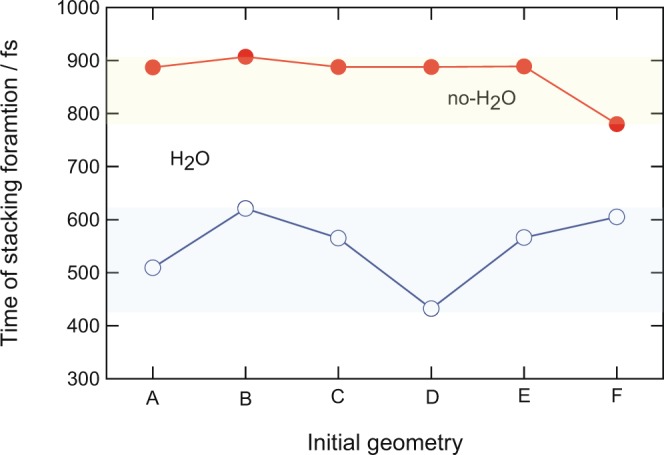
Time of π-stacking formation calculated by several initial structures. Basis sets used were (A) 6-311++G(2d,p), (B) 6-311++G(2d,2p), (C) 6-311++G(2df,2p), (D) 6-311++G(2df,2pd), (E) 6-311++G(3df,2pd), and (F) 6-311++G(3df,3pd).

In the previous sections, it was shown that the water molecule accelerated the π-stacking formation in (Bz)_2_^+^. However, the results were obtained from only one trajectory for the (Bz)_2_ and (Bz)_2_-H_2_O systems. In this section, the effect of the initial geometry on the time of π-stacking formation was investigated in detail. Six levels of theory were examined in the geometry optimizations. The geometry optimizations were carried out using A: 6-311++G(2d,p), B: 6-311++G(2d,2p), C: 6-311++G(2df,2p), D: 6-311++G(2df,2pd), E: 6-311++G(3df,2pd), and F: 6-311++G(3df,3pd) basis sets and the results are presented in Fig. 5. In the case of *no-H*_2_*O*, the time of π-stacking formation was calculated to be 780–907 fs, while the time was distributed in the 432–621 fs range in *H*_2_*O*. The wide distribution in H_2_O occurred because the position of H_2_O on (Bz)_2_ was slightly dependent on the basis sets used in the geometry optimizations. All levels of theory indicated that H_2_O significantly accelerated the time of π-stacking formation.

The initial geometries were also generated by thermal activation at 10 K. First, the geometries of the neutral dimer (Bz)_2_ and complex (Bz)_2_-H_2_O were fully optimized at the CAM-B3LYP/6-311++G(d,p) level. From these geometries, direct AIMD calculations of the neutral systems were carried out at constant temperature conditions at the CAM-B3LYP/6-311++G(d,p) level. The structures of (Bz)_2_ and (Bz)_2_-H_2_O fluctuated slightly around the equilibrium structures under this thermal condition (10 K). Twelve geometries were selected from each direct AIMD calculation of the cation state, which were performed at the CAM-B3LYP/6-31G(d) level. The results are given in Table 3 (10 K distribution). The times of π-stacking formation were calculated to be 457 fs (*H*_2_*O*) and 691 fs (*no-H*_2_*O*). From thermal sampling calculations, it was also found that H_2_O accelerated the π-stacking formation.

**Table 3:** Effects of initial geometries and levels of theory used in the calculations on the time of π-stacking formation (in fs) in (Bz)_2_^+^ (*no-H*_2_*O*) and (Bz)_2_^+^-H_2_O (*H*_2_*O*). Abbreviation “initial geom.” indicates the level of theory used in the geometry optimization of the neutral system. This structure was used as the initial geometry used in the direct AIMD calculation at time zero.

direct AIMD	initial geom.	*H*_2_*O*/fs	*no-H*_2_*O*/fs
cam-631 [a]	cam++G [b]	594	922
cam-cc-pVDZ [c]	cam++G	481	1068
10 K distribution	cam++G	457 (average)	691 (average)

[a] cam-631: CAM-B3LYP/6-31G(d).

[b] cam++G: CAM-B3LYP/6-311++G(d,p).

[c] cam-cc-pVDZ: CAM-B3LYP/cc-pVDZ.

To check the methodology dependence on the accelerating effect of H_2_O, direct AIMD calculations were carried out using a cc-pVDZ basis set with the CAM-B3LYP/ 6-311++G(d,p) optimized geometry. The results are given in Table 3 (cam-cc-pVDZ). The times of π-stacking formation were calculated to be 481 fs (*H*_2_*O*) and 1068 fs (*no-H*_2_*O*). From all the calculations, it was concluded that H_2_O accelerates the π-stacking formation in the benzene dimer cation.

### π-Stacking formation in (Bz)_3_-H_2_O^+^

#### Snapshots

**Figure 6:**
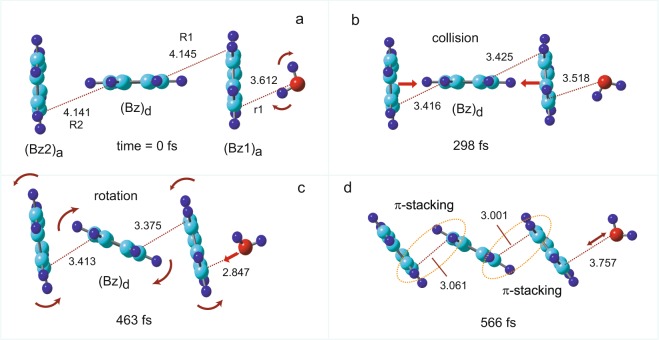
Snapshots of (Bz)_3_^+^-H_2_O after vertical ionization from the neutral state calculated as a function of time (intermolecular distances are in Å). The direct AIMD calculation was performed at the CAM-B3LYP/6-31G(d) level.

Figure 6 shows the snapshots of (Bz)_3_^+^-H_2_O following the ionization of (Bz)_3_-H_2_O. The optimized structure obtained at the CAM-B3LYP/6-311++G(d,p) level was used as the initial structure in the direct AIMD calculation (0 fs, point a). The intermolecular distances between the benzene molecules were 4.145 Å for R1 and 4.141 Å for R2, and the (Bz1)_a_-H_2_O distance (r_1_) was 3.612 Å. After ionization, both benzene molecules in the wing sites of (Bz1)_a_ and (Bz2)_a_ gradually approached (Bz)_d_ and collided at 298 fs (point b). After the collision, the three benzene rings rotated with respect to each other. The arrows schematically indicate the direction of rotation. At 463 fs, the intermolecular distances were R1 = 3.375 Å and R2 = 3.413 Å, and the structure of (Bz)_3_^+^ gradually approached the π-stacking form. At 566 fs (point d), the π-stacking form was complete. The behavior of H_2_O around (Bz)_3_^+^ was very similar to that of *n* = 2 (benzene dimer cation).

The snapshots of (Bz)_3_^+^ without H_2_O following the ionization of (Bz)_3_ are shown in Fig. S2. The reaction dynamics of (Bz)_3_^+^ were very similar to those of the benzene dimer cation (Fig. 3). The time of π-stacking formation was calculated to be 1155 fs in (Bz)_3_^+^ (*no-H*_2_*O*).

#### Potential energies

**Figure 7:**
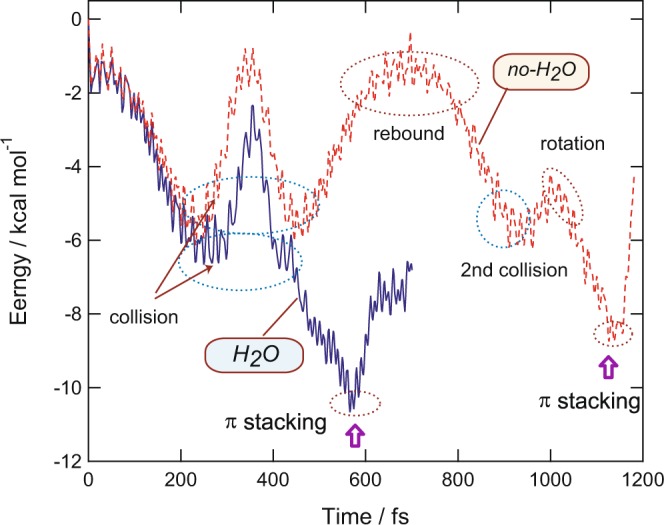
Time evolution of the potential energies of (Bz)_3_^+^ (*no-H*_2_*O*) and (Bz)_3_^+^-H_2_O (*H*_2_*O*). The direct AIMD calculations were performed at the CAM-B3LYP/6-31G(d) level.

The time evolution curves of the potential energy of (Bz)_3_^+^-H_2_O and (Bz)_3_^+^, following the ionization of the parent neutral complex, are shown in Fig. 7. After the ionization of (Bz)_2_-H_2_O, the potential energy decreased gradually, and the collision of (Bz1)_a_ and (Bz2)_a_ to (Bz)_d_ occurred at 298 fs. After the collision, the three benzene rings rotated with respect to each other, and π-stacking formation was complete at 566 fs. In the case of (Bz)_3_^+^, the π-stacking formation was complete at 1155 fs. Thus, the H_2_O molecule significantly accelerated the π-stacking formation in the benzene trimer cation as well as in the benzene dimer. The other levels of theory, i.e., the CAM-B3LYP/6-311++G(2d,p) and 6-311++G(2d,2p) optimized structures, gave similar results (Table 4).

**Table 4:** Effect of initial geometry on the time of π-stacking formation (in fs) in (Bz)_3_^+^ (*no-H*_2_*O*) and (Bz)_3_^+^-H_2_O (*H*_2_*O*). Abbreviation “initial geom.” indicates the initial geometry used in the direct AIMD calculation at time zero.

direct AIMD	initial geom.	*H*_2_*O*/fs	*no-H*_2_*O*/fs
cam-631 [a]	**cam**++**G** [b]	566	1155
cam-631	**cam-(2d,p)** [c]	567	1075
cam-631	**cam-(2d,2p)** [d]	520	1607

[a] cam-631: CAM-B3LYP/6-31G(d).

[b] cam++G: CAM-B3LYP/6-311++G(d,p).

[c] cam-(2d,p): CAM-B3LYP/6-311++G(2d,p).

[d] cam-(2d,2p): CAM-B3LYP/6-311++G(2d,2p).

## Discussion

### Reaction model

**Figure 8:**
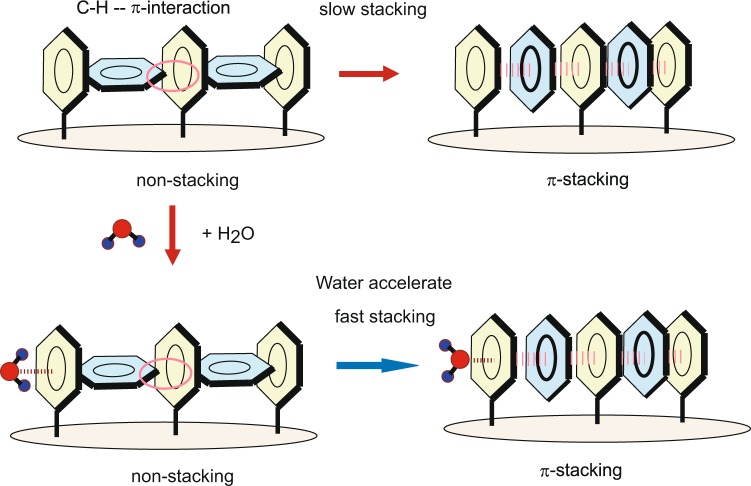
Schematic illustration of a model of the on-off switching element composed of a benzene cluster and the accelerating effect of H_2_O on the timescale of π-stacking formation.

Based on the results derived from the calculations presented above, a model was proposed for the effect of H_2_O on the timescale of the ON-OFF switching element composed of a benzene cluster. Figure 8 shows a schematic illustration of the proposed model. In the neutral state (upper figure), the C-H-π interaction is dominant between the benzene molecules, and the benzene cluster forms a non-stacking T-shape structure. After hole capture, the structure drastically changes from non-stacking to π-stacking forms. The hole can easily move along the stacked benzene rings. When the π-stacking form captures an electron, the structure spontaneously returns to the non-stacking form. If a water molecule interacts with a benzene molecule, the stacking rate is significantly accelerated because of the asymmetry of the electronic structure in the benzene rings. Nowadays, the surface functionalized graphenes have been designed and synthesized as molecular devices^30,31^. The present model would be applied to these molecular systems in near future. Also, the present investigation has open a field of the small cluster electronic devices.

### Concluding remarks

In the present calculations, several assumptions were employed in the ab initio calculations. First, the CAM-B3LYP functional was used in the direct AIMD calculation, and subsequently, the π-stacking reactions were discussed.

To check the functional dependence on reaction time, several functionals were examined in the direct AIMD calculations. The results are given in Table 5 (benzene dimer, *n* = 2) and 6 (benzene trimer, *n* = 3). The direct AIMD calculations with the APFD functional showed that the time of π-stacking was 358 fs (*H*_2_*O*) and 883 fs (*no-H*_2_*O*) for *n* = 2. In the case of *n* = 3, these times were calculated to be 434 fs (*H*_2_*O*) and 1005 fs (*no-H*_2_*O*). All results shown in Tables 5 and 6 suggest that H_2_O accelerates the π-stacking formation in both benzene dimer and trimer cation.

**Table 5:** Effect of the functional used in the direct AIMD calculations on the time of π-stacking formation (in fs) in (Bz)_2_^+^ (*no-H*_2_*O*) and (Bz)_2_^+^-H_2_O (*H*_2_*O*). The CAM-B3LYP/6-311++G(d,p) optimized geometries were used as the initial geometries at time zero. The direct AIMD calculations were carried out using 6-31G(d) basis set.

Functional	*H*_2_*O*/fs	*no-H*_2_*O*/fs
CAM-B3LYP	594	922
APFD	358	883
M062X	436	871
B3LYP	459	1223
M052X	452	834

**Table 6:** Effect of the functional used in the direct AIMD calculations on the time of π-stacking formation (in fs) in (Bz)_3_^+^ (*no-H*_2_*O*) and (Bz)_3_^+^-H_2_O (*H*_2_*O*). The CAM-B3LYP/6-311++G(d,p) optimized geometries were used as the initial geometries at time zero. The direct AIMD calculations were carried out using 6-31G(d) basis set.

Functional	*H*_2_*O*/fs	*no-H*_2_*O*/fs
CAM-B3LYP	566	1155
APFD	434	1005
M062X	654	897

As for the other factors, the position of H_2_O around neutral (Bz)_n_ (*n* = 2 and 3), methanol-(Bz)_n_ (instead of H_2_O), and zero-point vibration were also examined (supporting information).

## Conclusion

The calculations presented herein revealed that a H_2_O molecule accelerates the time of π-stacking formation in a benzene molecular system. The times of stacking formation in the benzene dimer (*n* = 2) and trimer (*n* = 3) cations were calculated to be 594 fs (*H*_2_*O*) and 922 fs (*no-H*_2_*O*), and 566 fs (*H*_2_*O*) and 1155 fs (*no-H*_2_*O*), respectively. Thus, H_2_O showed a positive effect in benzene-based molecular electronics. This tendency was not dependent on the level of theory used for calculations. The acceleration primarily originated from the re-orientation of H_2_O on benzene cluster cation following the hole capture.

While previous studies have shown that H_2_O suppresses the conductance and destroys the circuit of electron transport in molecular electronics (i.e., has negative effects)^14–20^, the present study demonstrated that H_2_O produces positive effects in benzene-based molecular electronics.

## Supplementary information

supporting_information
