# Supplementary material for: Water-accelerated π-Stacking Reaction in Benzene Cluster Cation
Source: Sci Rep. 2019 Feb 20;9:2377. doi: 10.1038/s41598-019-39319-7 (PMC6382828; doi:10.1038/s41598-019-39319-7)
Supplement: Supplementary file 1 — supporting_information [file 41598_2019_39319_MOESM1_ESM.pdf]

SUPPORTING INFORMATION

Water-accelerated π-Stacking Reaction in Benzene Cluster Cation

Hiroto TACHIKAWA*, Ryoshu IURA and Hiroshi KAWABATA

Division of Applied Chemistry, Graduate School of Engineering,

Hokkaido University, Sapporo 060-8628, JAPAN

1. Spin densities

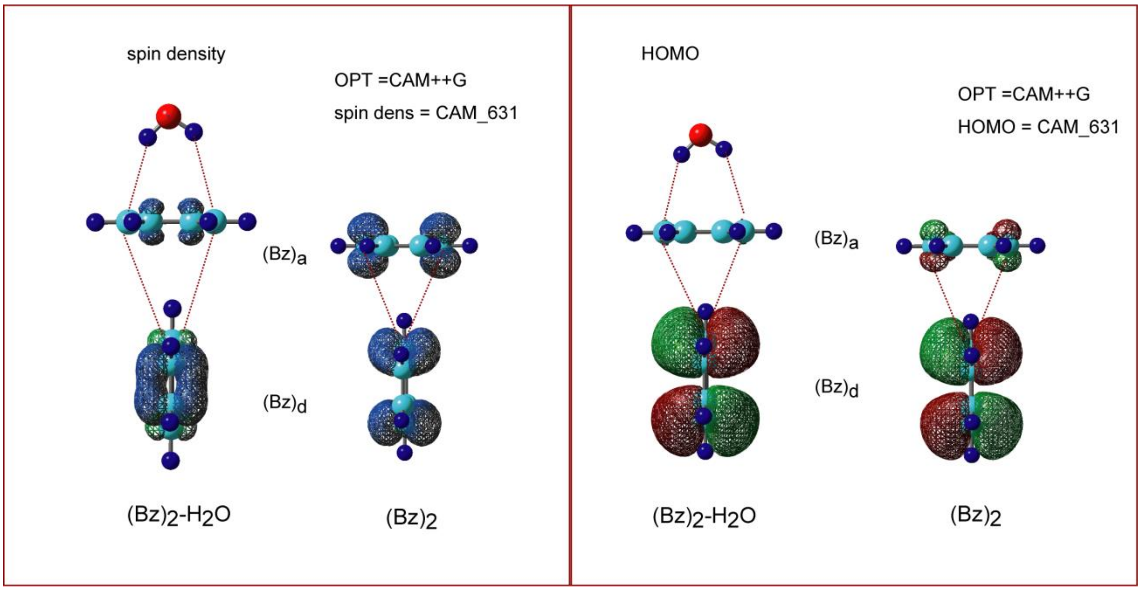

Figure S1. (Left) Spatial distribution of spin densities on cation radicals of benzene dimer and hydrated benzene dimer at vertical ionization points from the neutral states. (Right) HOMOs of benzene dimer and hydrated benzene dimer (neutral state). The calculation was carried out at the CAM-B3LYP/6-311++G(d,p) level.

2. Snapshots of $(Bz)_3^+$

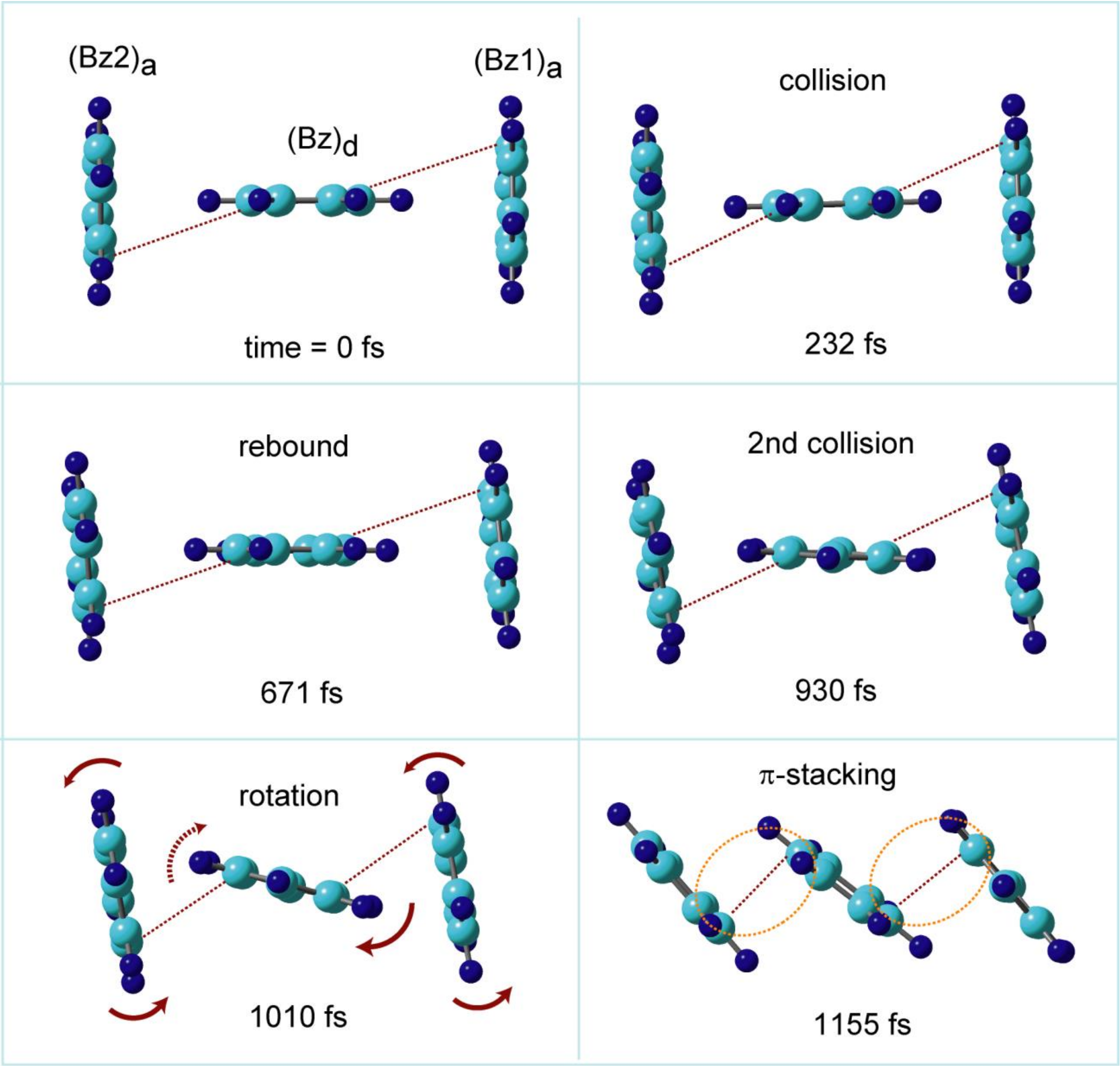

Figure S2. Snapshots of benzene trimer cation after vertical ionization from neutral state calculated as a function of time. The direct AIMD calculation was carried out at the CAM-B3LYP/6-31G(d) level. The π-stacking was completed at time= 1155 fs.

3. π-Stacking reaction in $CH_3OH\text{-}(Bz)_2^+$.

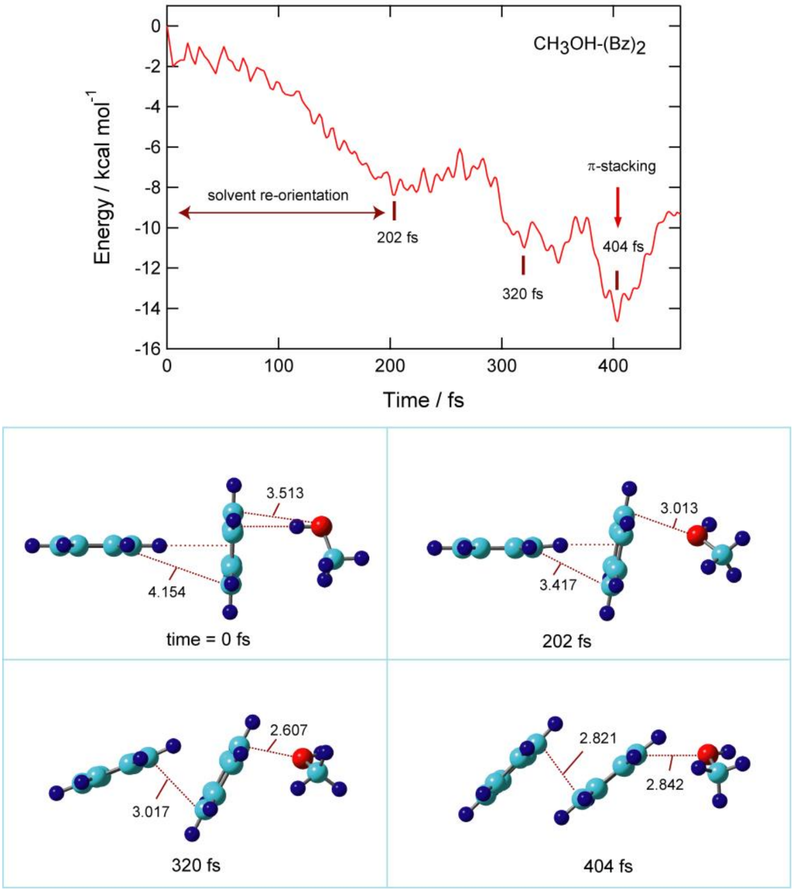

Figure S3. Results of direct AIMD calculation of $(Bz)_2^+$-methanol molecule (CH_3OH) following the ionization of the parent neutral species. **(Upper):** Time evolution of the potential energy of $(Bz)_2^+\text{-}CH_3OH$. **(Lower):** Snapshots of $(Bz)_2^+$- CH_3OH after vertical ionization from the neutral state calculated as a function of time (intermolecular distances are in Å). The direct AIMD calculation was performed at the CAM-B3LYP/6-31G(d) level. The structure of $(Bz)_2^+\text{-}CH_3OH$ was optimized at the CAM-B3LYP/6-311++G(d,p) level.

4. The other optimized structures of $(Bz)_n$-H_2O (n= 2 and 3)

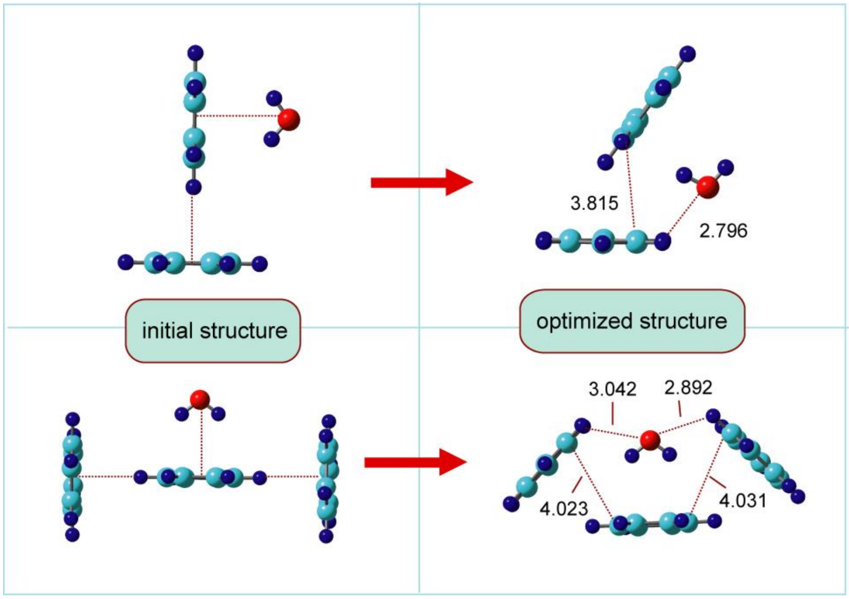

Figure S4. Initial and final structures of the benzene dimer-water complex, $(Bz)_2$-H_2O, and benzene trimer-water complex $(Bz)_3$-H_2O calculated at the CAM-B3LYP/6-311++G(d,p) level. The values indicate the intermolecular distances (in Å).

5. H_2O-Position dependence in p-stacking reaction of $(Bz)_2^+$.

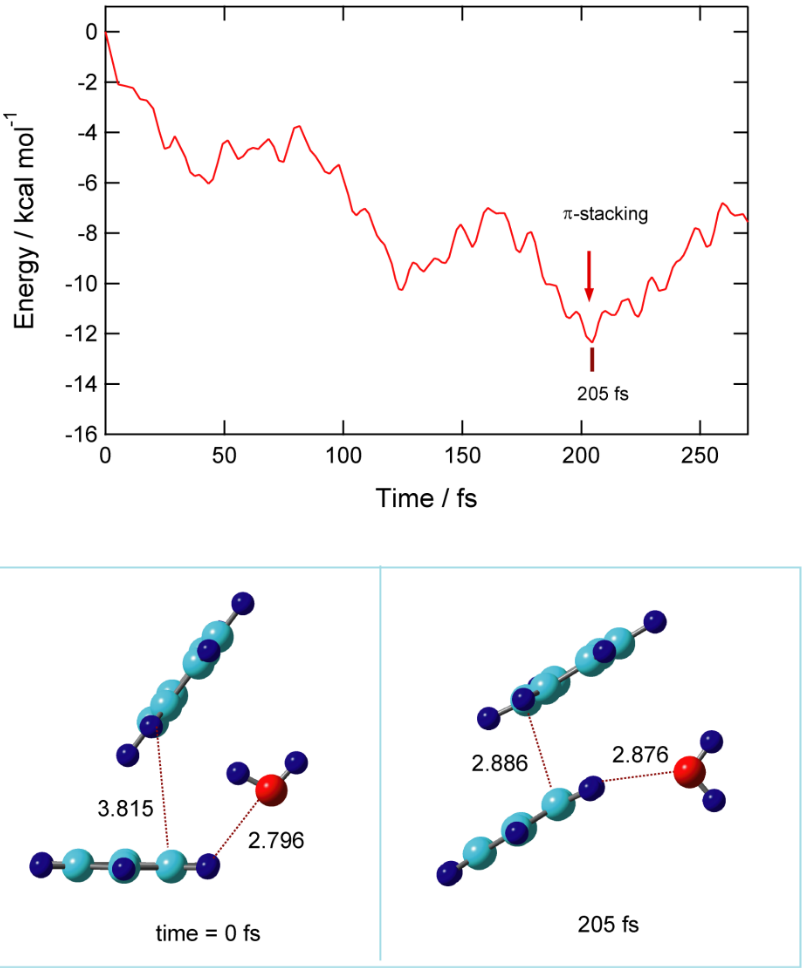

Figure S5. Results of direct AIMD calculation of $(Bz)_2^+$-H_2O following the ionization of the parent neutral species. **(Upper):** Time evolution of the potential energy of $(Bz)_2^+$-H_2O. **(Lower):** Snapshots of $(Bz)_2^+$-H_2O after vertical ionization from the neutral state calculated as a function of time (intermolecular distances are in Å). The direct AIMD calculation was performed at the CAM-B3LYP/6-31G(d) level.

6. H_2O-Position dependence in p-stacking of $(Bz)_3^+$.

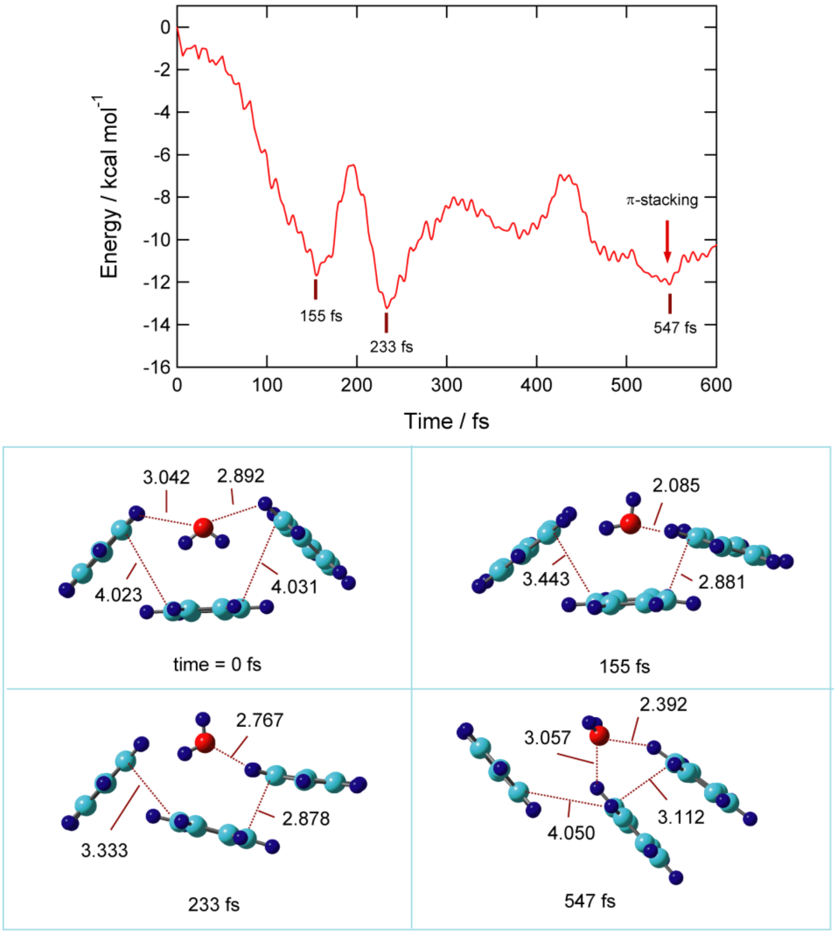

Figure S6. Results of direct AIMD calculation of $(Bz)_3^+$-H_2O following the ionization of the parent neutral species. **(Upper):** Time evolution of the potential energy of $(Bz)_3^+$-H_2O. **(Lower):** Snapshots of $(Bz)_3^+$-H_2O after vertical ionization from the neutral state calculated as a function of time (intermolecular distances are in Å). The direct AIMD calculation was performed at the CAM-B3LYP/6-31G(d) level.

7. Potential energy curves

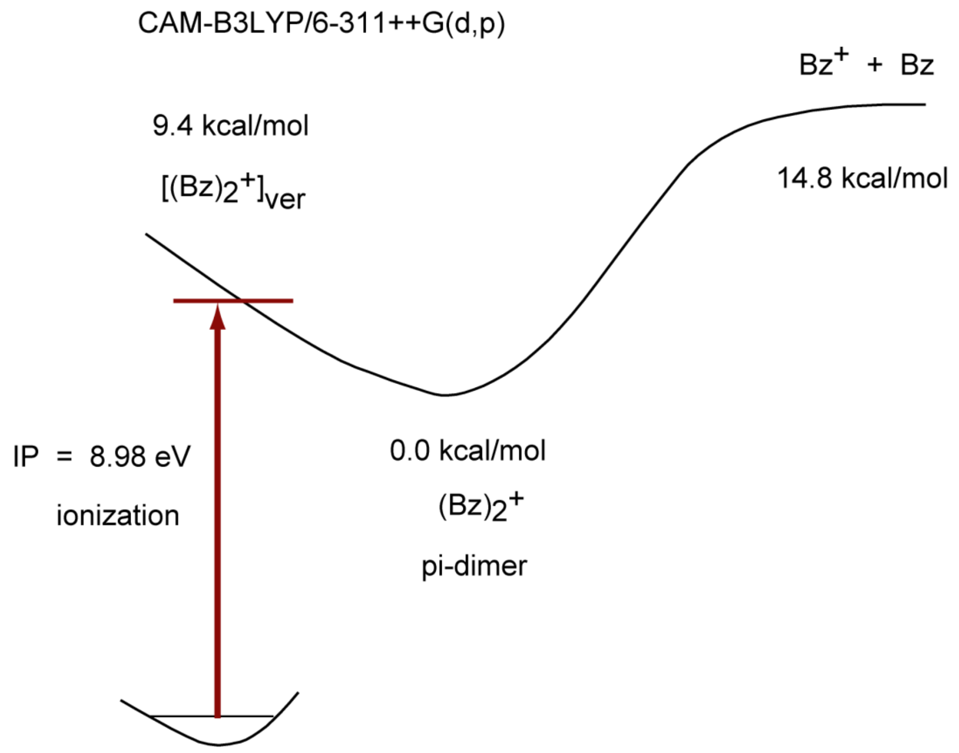

Figure S7. Schematic illustration of potential energy curves for benzene dimer system. Values are relative energies in kcal/mol. The calculation was performed at the CAM-B3LYP/6-311++G(d,p) level.

8. Reaction rates

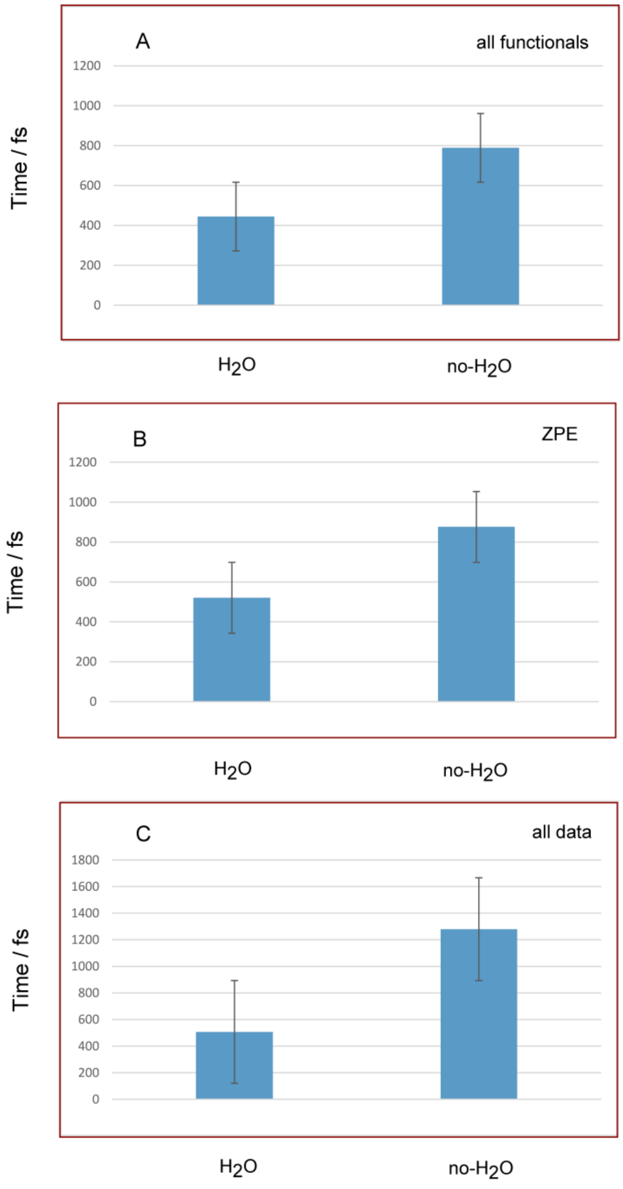

Figure S8. Schematic illustration of potential energy curves for benzene dimer system. Values are relative energies in kcal/mol. The calculation was performed at the CAM-B3LYP/6-311++G(d,p) level.
